# A mutual comparison of pregnancy outcomes between different conception modes: a propensity score matching based retrospective cohort study

**DOI:** 10.3389/fendo.2024.1351991

**Published:** 2024-01-25

**Authors:** Chang-Fa Sun, Jian-Zhong Sheng, He-Feng Huang

**Affiliations:** ^1^ Centre for Reproductive Medicine, Shanghai East Hospital, School of Medicine, Tongji University, Shanghai, China; ^2^ International Institutes of Medicine, the Fourth Affiliated Hospital, Zhejiang University School of Medicine, Yiwu, China; ^3^ International Peace Maternity and Child Health Hospital, School of Medicine, Shanghai Jiao Tong University, Shanghai, China; ^4^ Obstetrics and Gynecology Hospital, Institute of Reproduction and Development, Fudan University, Shanghai, China; ^5^ Obstetrics and Gynecology Hospital, Key Laboratory of Reproductive Genetics (Ministry of Education), School of Medicine, Zhejiang University, Hangzhou, China

**Keywords:** OI, art, preterm birth, low birth weight, gestational diabetes

## Abstract

**Background:**

Assisted reproductive technology (ART) has been reported to have negative effects on maternal and neonatal health. Ovulation induction (OI) was reported to be associated with alteration of epigenetic modification of mice embryos, and extinguishing the influence of ovulation induction and *in vitro* operations on maternal and neonatal health will bring benefits for reducing side effects. The present study aimed to determine whether ovulation induction alone and ART are associated with adverse pregnancy outcomes and whether ART could induce a higher risk than ovulation induction alone.

**Methods:**

A total of 51,172 cases with singleton live birth between Jan 2016 and May 2019 at the International Peace Maternal and Child Health Hospital were included in this study. Conception modes documented during registration were classified into natural conception (NC), OI, and ART. Pregnancy outcomes of the three groups with balanced baseline characteristics by propensity score matching were compared. The relative risks of maternal and neonatal outcomes were calculated by logistic regression analysis.

**Results:**

Compared with natural conception, infertility treatments are associated with gestational diabetes (OI: OR 1.72, 95% CI 1.31-2.27; ART: OR 1.67, 95% CI 1.26-2.20), preeclampsia/eclampsia (OI: OR 1.86, 95% CI 1.03-3.36; ART: OR 2.23, 95% CI 1.26-3.92). Even if gestational diabetes, gestational hypertension, and placental problems were adjusted, infertility treatments are associated with birth before 37 weeks (OI: OR 1.99, 95% CI 1.28-3.12; ART: OR 1.70, 95% CI 1.08-2.69), low birth weight (OI: OR 2.19, 95% CI 1.23-3.91; ART: OR 1.90, 95% CI 1.05-3.45), and SGA (OI: OR 2.42, 95% CI 1.20-4.87; ART: OR 2.56, 95% CI 1.28-5.11). ART but not OI is associated with a higher risk of birth before 34 weeks (OR:3.12, 95% CI 1.21-8.05). By comparing the OI group with the ART group, we only found that ART could induce a higher ratio of placental problems (5.0%, 26/518 vs 2.1%, 11/519, p<0.05).

**Conclusion:**

Both OI and ART are associated with adverse pregnancy outcomes. ART induced comparable negative effects with OI on gestational complications, birth weight, and premature birth (<37 weeks). However, ART resulted in a higher risk of placental problems than group NC and OI. The incidence of birth before 34 weeks of gestation in the ART group tends to be higher than in the OI group, but not statistically significant. The side effects of ART may originate from OI.

## 1 Introduction

Infertility impacts 8% to 12% of the childbearing population worldwide (1, 2), and the prevalence of infertility was up to 25% among couples of reproductive ages in China (3). The World Health Organization reported 1 in 6 people globally was affected by infertility (4), and now, assisted reproductive technology (ART) has become a dominant method for infertility treatment (5). However, ART has been reported to result in negative effects on maternal and neonatal health, and the safety of ART continues to be a matter of concern (6). An elevated risk of multiple births, which was associated with ART, has been thought to be one of the causes of ART-induced negative effects on pregnant women and neonates (7, 8). Although single embryo transfer is encouraged now (9), negative effects on singleton pregnancy could be observed (10, 11). The association between ART and a higher risk of low birth weight (LBW) and preterm birth (PTB) (10, 12, 13) has been well studied because of their fatal impacts on neonates (14). On the other hand, women conceived by ART were reported to have higher risks of pregnancy diabetes and hypertensive diseases (15).

Medications for ovulation induction (OI) were utilized in more than 95% of IVF cycles (16). Medications for follicle growth and ovulation are applied in both OI and ART. The dosage of the medications is lower in OI, and ART also includes more processes which are *in vitro* gametes combination, the culture of embryos for 3 to 5 days in dishes, and the transfer of embryos to the uterus of patients. Extinguishing the influence of OI and *in vitro* operations on maternal and neonatal health will bring benefits for reducing side-effects. A comprehensive comparison among natural conception, conception by OI, and conception by ART will help us to know whether OI alone could result in adverse impacts and whether *in-vitro* operations would add more risks to adverse pregnancy outcomes. Although some studies compared pregnancy outcomes between IVF and natural conception, few studies conducted mutual comparisons of pregnancy outcomes among OI, IVF, and natural conception.

In the present study, we compared maternal and neonatal outcomes among spontaneous conception, OI, and ART with balanced baseline characteristics through propensity score matching (PSM).

## 2 Materials and methods

### 2.1 Study design and participants

Pregnant women who registered at the International Peace Maternal and Child Health Hospital, and delivered between January 1, 2016, and May 31, 2019, were enrolled. The ethical approval for this study was granted by the Ethics Committee of the International Peace Maternity and Child Health Hospital (GLW 2017-81). Conception mode, maternal pre-pregnancy body mass index (pre-BMI), age, education level, gravity, race, gestational complications (including gestational diabetes, hypertensive disorder, eclampsia/preeclampsia, intrahepatic cholestasis of pregnancy, anemia, and placental problem), gestational age at delivery, neonatal birth weight, gender, neonatal disease diagnosis were drawn from the electronic medical record system. Cases with multiple pregnancies, missing conception mode, stillbirth, and artificial insemination (AI) were excluded. The population was classified into three groups based on conception modes: natural conception (NC), ovulation induction (OI), and assisted reproductive technology (ART). We stratified maternal age into <30 years, 30-34 years and ≥35 years; maternal pre-pregnancy BMI into <18.5 kg/m2, 18.5-23.9 kg/m2, 24-27.9 kg/m2 and ≥28 kg/m2; gravidity into 1, 2 and ≥3 times; education level into high school education or lower, university education and postgraduate education; race into Han Chinese and other. Placental problems include low-lying placenta, placenta accreta, battledore placenta, velamentous placenta and placenta succenturiate. According to the birthweight and gestational age, we defined preterm birth (PTB) as gestational duration before the 37th week of gestation, and PTB was classified into <32 weeks, <34 weeks and <37 weeks. Low birth weight (LBW) was defined as birth weight below 2500g and extremely LBW (eLBW) as birth weight below 1500 g. Small for gestational age (SGA) was defined as birth weight below the 10th percentile of the gestational age- and sex-specific birth weight reference according to the INTERGROWTH-21st.

### 2.2 Statistical methods

Statistical analyses were performed using SPSS version 24, and PSM was realized by EZR version 1.60. The categorical variables were presented as numbers and percentages, and the difference was calculated by chi-square test or fisher exact test. To make the maternal baseline balanced among groups, categories of maternal age, pre-BMI, maternal education and gravity were matched. A 1:1 matching between OI and ART was performed, and a subsequent 1:1 matching among NC and the former dataset (OI and ART) was performed. A single-variable logistic regression model was conducted to analyze the association between conception modes and pregnancy outcomes. Gestational diabetes, placental problems and gestational hypertensive disorders were further adjusted to analyze if conception modes impact neonatal outcomes via gestational complications. Statistical significance was set at p<0.05.

## 3 Results

**Figure 1:**
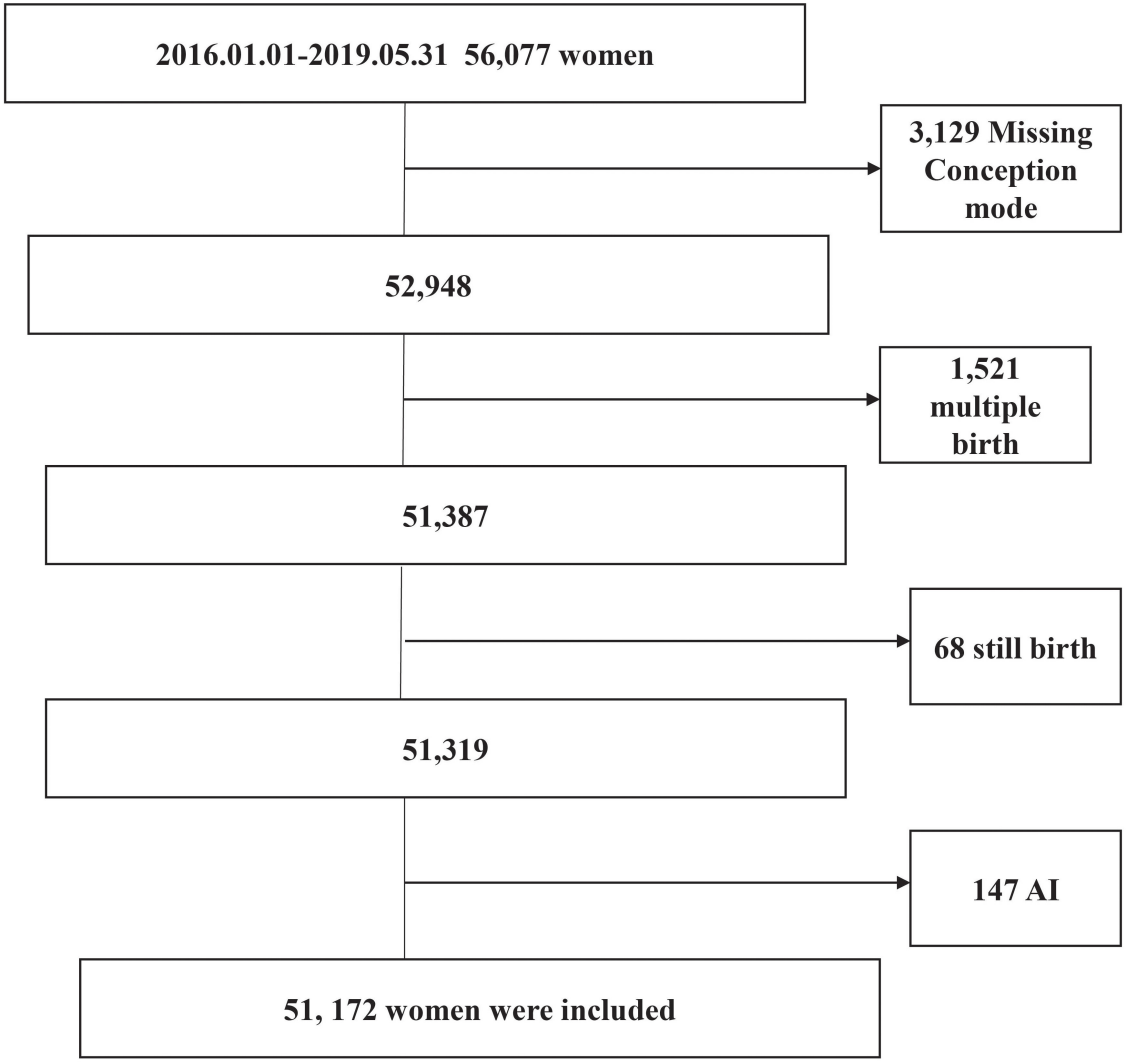
The Flow Diagram for Case Selection.

The details of 56,077 women who delivered between January 1, 2016 and May 31, 2019 were recorded in the electronic medical record system of the International Peace Maternal and Child Health Hospital. Of these, 3,129 women with missing conception mode, 1,521 with multiple births, 68 with stillbirths, and 147 conceived by artificial insemination were excluded (**Figure 1**). Finally, 51,172 pregnant women were included in this study, among whom 92.5% (47,348/51,172), 1.1% (539/51,172) and 6.4% (3,285/51,172) were conceived naturally, by ovulation induction and by assisted reproductive technology, respectively.

By analyzing the total of 51,172 included cases, there was a higher proportion of women with age <30 years in the OI group, while a higher proportion of women ≥35 years in the ART group. The ratios of pre-BMI ≥24, first pregnancy, GDM, hypertension, and preeclampsia/eclampsia were higher in both the OI group and the ART group. A higher ratio of placental problems and a lower ratio of university or postgraduate education were found in the ART group, but not in the OI group, compared to the NC group. The prevalence of anemia was lower in both the OI group and the ART group, compared to the NC group. The higher ratios of admission to the NICU, birth before 32 weeks of gestation and birth before 34 weeks of gestation were found in the ART group, but not in the OI group, compared with the NC group. Both the OI group and the ART group have higher ratios of birth before 37 weeks, birth weight lower than 1,500 g and birth weight lower than 2,500 g, compared to the NC group (**Supplementary Table 1**).

The total of 1,037 natural concepted women, 519 women conceived by ovulation induction and 518 women conceived by ART were matched ultimately according to the propensity score. After PSM, there were no statistically significant differences in the categories of maternal age, pre-BMI, gravidity, education level, anemia and Han Chinese among three groups. Compared to the NC group, the OI group had higher ratios of women with GDM, hypertension, preeclampsia/eclampsia, and neonates with delivery before 37 weeks of gestation, birth weight lower than 2,500 grams and SGA, respectively. Compared to the NC group, the ART group had higher ratios of women with placental problems, GDM, hypertensive disorders preeclampsia/eclampsia, and neonates with delivery before 34 weeks, delivery before 37 weeks, birth weight lower than 2,500 grams and SGA, respectively. By comparing the OI group with the ART group, only a higher ratio of placental problems was found in the ART group (**Table 1**).

**Table 1:** Maternal and neonatal characteristics after PSM.

Group		NC	OI		IVF		
		N=1,037	N=519	*p*	N=518	*p*	
		n (%)	n (%)	Compare to NC	n (%)	Compare to NC	Compare to OI
Maternal characteristics							
Age (y)	<30	434 (41.9)	217 (41.8)	ns	217 (41.9)	ns	ns
	30-34	515 (49.7)	258 (49.7)		257 (49.6)		
	≥35	88 (8.5)	44 (8.5)		44 (8.5)		
Pre-BMI (kg/m^2^)	<18.5	80 (7.7)	40 (7.7)	ns	40 (7.7)	ns	ns
	≥18.5<24	674 (65.0)	337 (64.9)		337 (65.1)		
	≥24<28	144 (13.9)	72 (13.9)		72 (13.9)		
	≥28	68 (6.6)	34 (6.6)		34 (6.6)		
	missing	71 (6.8)	36 (6.9)		35 (6.8)		
Gravidity	1	612 (59.0)	306 (59.0)	ns	306 (59.1)	ns	ns
	2	238 (23.0)	119 (22.9)		119 (23.0)		
	≥3	179 (17.3)	90 (17.3)		89 (17.2)		
	missing	8 (0.8)	4 (0.8)		4 (0.8)		
Education level	≤high school	75 (7.2)	38 (7.3)	ns	37 (7.1)	ns	ns
	university education	796 (76.8)	398 (76.7)		398 (76.8)		
	postgraduate education	166 (16.0)	83 (16.0)		83 (16.0)		
Han Chinese		1022 (98.6)	512 (98.7)	ns	510 (98.5)	ns	ns
Anemia		320 (30.9)	161 (31.0)	ns	151 (29.2)	ns	ns
GDM		141 (13.6)	111 (21.4)	<0.001	108 (20.8)	<0.001	ns
Hypertension		69 (6.7)	54 (10.4)	<0.05	51 (9.8)	<0.05	ns
ICP		5 (0.5)	3 (0.6)	ns	3 (0.6)	ns	ns
Preeclampsia/eclampsia		24 (2.3)	22 (4.2)	<0.05	26 (5.0)	<0.05	ns
Placental problems		22 (2.1)	11 (2.1)	ns	26 (5.0)	<0.01	<0.05
Neonatal characteristics							
Male		510 (49.2)	254 (48.9)	ns	270 (52.1)	ns	ns
Jaundice		16 (1.5)	11 (2.1)	ns	14 (2.7)	ns	ns
NICU		12 (1.2)	6 (1.2)	ns	9 (1.7)	ns	ns
Preterm birth	<32 weeks	4 (0.4)	3 (0.6)	ns	7 (1.4)	ns	ns
	<34 weeks	7 (0.7)	7 (1.3)	ns	12 (2.3)	<0.05	ns
	<37 weeks	42 (4.1)	42 (8.1)	<0.01	38 (7.3)	<0.01	ns
Low birth weight	<1500 g	3 (0.3)	5 (1.0)	ns	4 (0.8)	ns	ns
	< 2500 g	23 (2.2)	26 (5.0)	<0.01	23 (4.4)	<0.01	ns
SGA		15 (1.4)	18 (3.5)	<0.05	19 (3.7)	<0.01	ns

Ns, no significance; GDM, Gestational diabetes mellitus; ICP, intrahepatic cholestasis of pregnancy; NICU, neonatal intensive care unit; SGA, small for gestational age.

**Figure 2:**
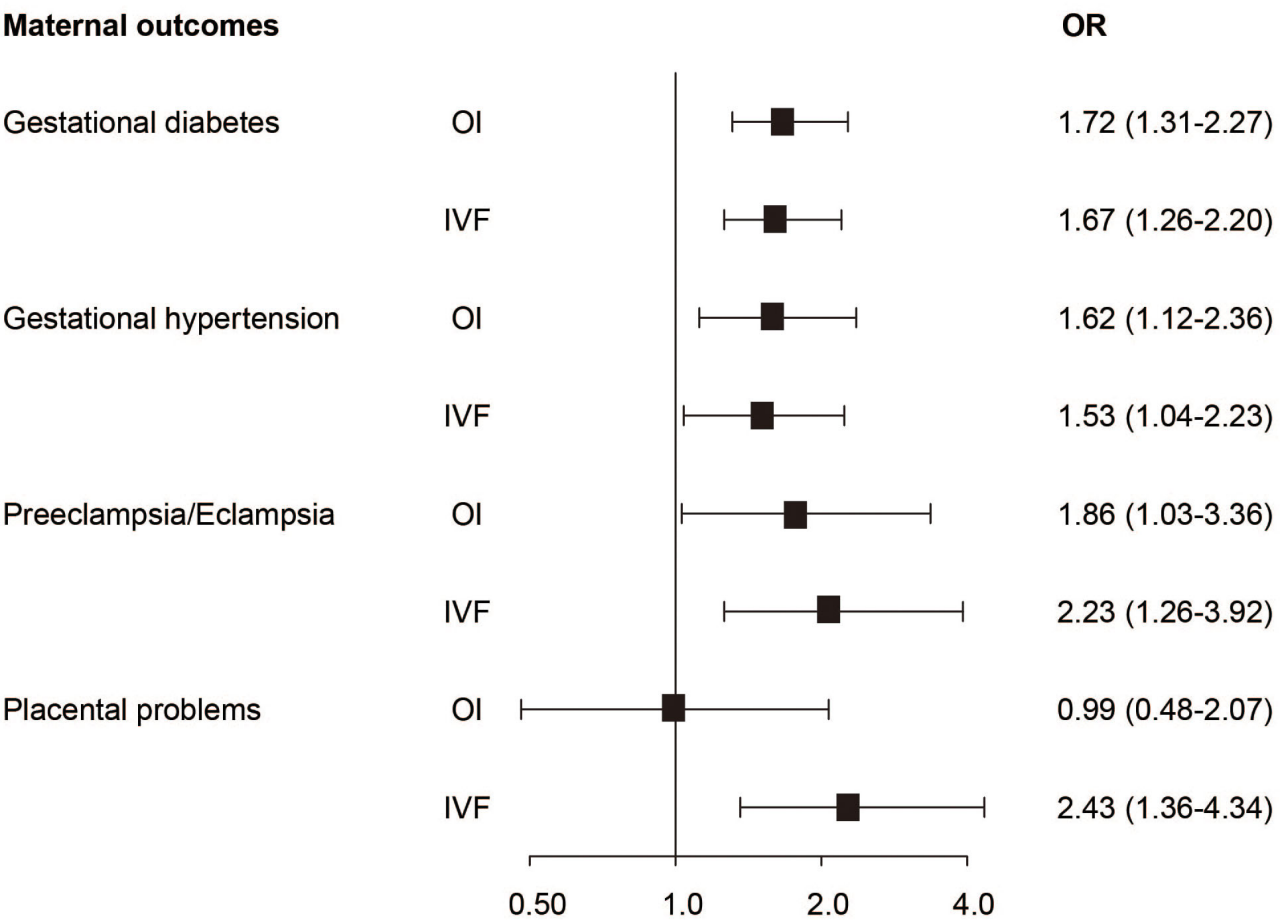
The association between conception mode and maternal outcomes.

**Figure 3:**
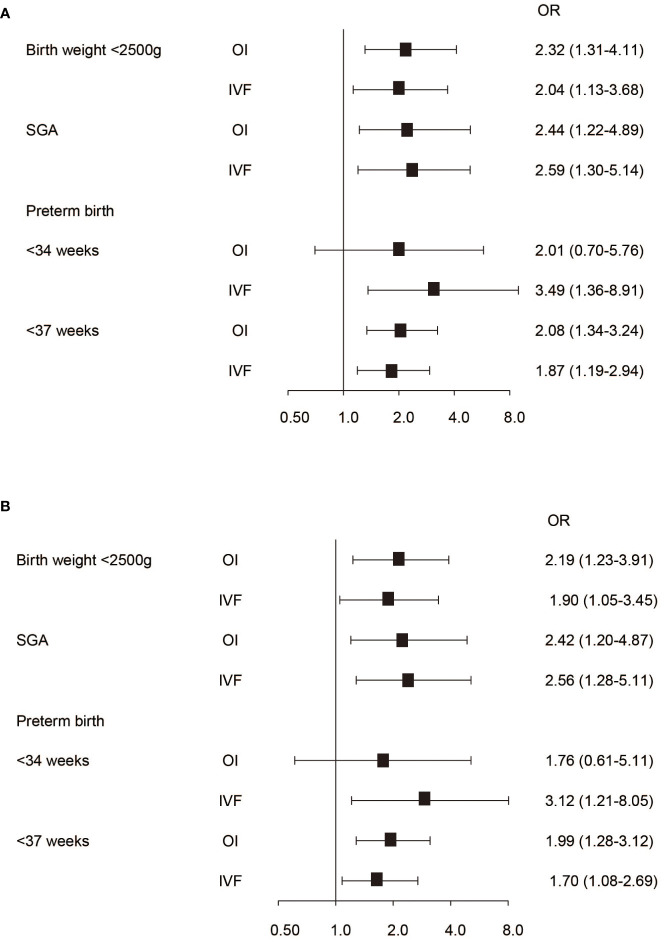
The association between conception mode and neonatal outcomes. **(A)** Neonatal outcomes without adjusting pregnancy complications. **(B)** Neonatal outcomes with adjusting pregnancy complications.

Compared to the NC group, women in the OI group had a 1.72-fold risk of GDM, a 1.62-fold risk of hypertensive disorders and a 1.86-fold risk of preeclampsia/eclampsia, respectively (**Figure 2**). Neonates in the OI group had a 2.32-fold risk of birth weight lower than 2,500g, a 2.08-fold risk of birth before 37 weeks of gestation and a 2.44-fold risk of SGA, respectively (**Figure 3A**). Women conceived by ART had a 1.67-fold risk of GDM, a 1.53-fold risk of hypertension, a 2.23-fold risk of preeclampsia/eclampsia and a 2.43-fold risk of placental problems, respectively (**Figure 2**). Neonates in the ART group had a 2.04-fold risk of birth weight lower than 2,500 grams, a 3.49-fold risk of birth before 34 weeks of gestation, a 1.7-fold risk of delivery before 37 weeks of gestation and a 2.59-fold risk of SGA, respectively (**Figure 3A**). Even if hypertension, GDM, and placental diseases were adjusted the associations were also significant (**Figure 3B**).

## 4 Discussion

### 4.1 Main findings

This cohort study compared maternal and neonatal outcomes in pregnant women conceived naturally, by OI alone and by ART. In the present study, 1.1% of neonates were born after OI, and 6.4% of neonates were born after ART. By logistic regression analysis of the included cases, we found that both OI alone and ART were associated with higher risks of preterm birth, low birth weight, SGA, gestational diabetes, gestational hypertensive disorders, and preeclampsia/eclampsia than the NC group. Compared to the OI group, the ART group had a higher risk of placental problems but no other worse outcomes. The adverse neonatal outcomes were independent of placental problems, GDM, and gestational hypertensive disorders.

The comparison of obstetric and neonatal outcomes between IVF and NC has been well investigated. Increased risks of gestational hypertensive disorders, low birth weight, small for gestational age, and gestational diabetes were found (15, 17). Our former study demonstrated that the concentration of serum estrogen after fresh embryo transfer was significantly higher than frozen embryo transfer and was associated with an increased risk of SGA (18). Usal et al. (19) found superovulation induced higher expression of DNMT proteins in mouse oocytes and early-stage embryos. Tang et al. (20) found repeated superovulation in mice induced a reduction of the acetylation level of histone 4 at lysine 12 (H4K12ac) and histone 4 at lysine 16 (H4K16ac) in early embryos. By single-cell DNA methylation sequencing, a study found superovulation was associated with differential DNA methylation related to glucose metabolism, cell cycle, and embryo implantation (21). However, few studies determined the impacts of OI alone on pregnancy outcomes and whether the *in vitro* operations in IVF would bring more serious consequences. A former study that compared neonatal outcomes between NC and OI alone showed that OI was associated with LBW and PTB, but the differences were reduced or disappeared after the adjustment of confounding factors (22). Another study stratified conception mode into NC, medication only, and ART (23).Compared to the NC group, both our study and the former study found a higher risk of SGA in the OI group, 2.4-fold and 1.7-fold respectively; and a higher risk of SGA in the ART group, 2.5-fold and 1.9-fold respectively. For the definition of SGA, they referred to criteria in white and black infants, and we referred to the latest criteria from the project INTERGROWTH-21st. Compared to the NC group, both our study and the former study found a higher risk of birth before 37 weeks of gestation, 1.7-fold and 1.9-fold respectively; we found a 1.9-fold risk of birth before 37 weeks in the OI group, but there is no statistical difference in their study. This may be due to different races of pregnant women and survey times among our investigations because the general incidence of premature birth was obviously higher in the former study. A few studies classified OI and AI into a non-ART group, and compared neonatal outcomes between natural conception, non-ART treatment, and ART treatment (10, 11). Wang et al. (10) found both ART and non-ART treatment could induce a higher risk of PTB compared to natural conception. Stern et al. (11) compared pregnancy outcomes between the groups with detailed stratification of infertility diagnosis, and found that both ART and non-ART treatment induced a higher risk of PTB in all subgroups, including tubal, PCOS, other ovulatory and endometriosis-related infertility. Compared with NC, non-ART and ART treatments were found to be associated with increased risks of pregnancy hypertension, pregnancy diabetes, eclampsia/preeclampsia and low birth weight in most subgroups. However, unlike these studies, we only found ART was associated with a higher risk of placental problems but no other worse outcomes than ovulation induction alone.

### 4.2 Strengths and limitations

Although this study was conducted in one hospital, the pregnant women included in our study were from different provinces of China, and the fertility treatments were applied in different fertility centers. The results by analyzing the matched database are generally in accordance with the original database. Although the underlying infertility diagnosis unavailable, PSM was performed in this study to reduce the selection bias (24). A mutual comparison among three groups with balanced characteristics is a characteristic feature of the present study. There are also several limitations to this study. We do not have information on the causes of infertility, and some infertility-related primary diseases may be an important factor for adverse pregnancy outcomes. We also do not have information on whether and which kinds of medications were used in the procedure of ART. Last but not least, the conception mode was self-reported by the pregnant women, and we can’t verify the authenticity of the information. A multicenter investigation in further study with serum hormone levels, causes of infertility, fresh or frozen embryo transfer, and the protocols for ovulation induction will make the conclusion more solid.

### 4.3 Interpretation

Different from the prior studies, we found that ART would not increase risks of pregnancy diabetes, pregnancy hypertension, LBW and PTB, compared with ovulation induction alone. Our results suggest that the security of ART and OI is nearly equivalent and medications for ovulation may be the origin of adverse outcomes. We think a potentially higher ratio of frozen embryo transfer in our cohort may be the cause of inconsistency with former studies. A series of evidence in recent years suggests frozen embryo transfer could bring better pregnancy outcomes by avoiding excess physiological hormone exposure than fresh embryo transfer (25, 26). Although the database does not record the information on transferred embryos, a former cohort study in Shanghai reported that frozen embryo transfer accounted for 68.3% (4,071/5,960) from 2013 to 2018 (27). A former study compared pregnancy outcomes between different controlled ovulation stimulation protocols in IVF (28). Compared to natural IVF cycles, medications-induced ovulation is associated with more severe consequences. However, the study also cannot demonstrate that medications for controlled ovulation alone could result in worse pregnancy outcomes than natural conception.

## 5 Conclusion

Both OI and ART are associated with adverse pregnancy outcomes. ART induced comparable negative effects with OI on gestational complications, birth weight, and premature birth (<37 weeks). However, ART resulted in a higher risk of placental problems than group NC and OI. The incidence of birth before 34 weeks of gestation in the ART group tends to be higher than the OI group, but not statistically significant. The side effects of ART may originate from OI.

## Data availability statement

The raw data supporting the conclusions of this article will be made available by the authors, without undue reservation.

## Ethics statement

The studies involving humans were approved by the Ethics Committee of the International Peace Maternity and Child Health Hospital. The studies were conducted in accordance with the local legislation and institutional requirements. Written informed consent for participation was not required from the participants or the participants’ legal guardians/next of kin in accordance with the national legislation and institutional requirements.

## Author contributions

CS: Methodology, Software, Writing – original draft. JS: Supervision, Writing – review & editing. HH: Supervision, Writing – review & editing.
