# Supplementary material for: A mutual comparison of pregnancy outcomes between different conception modes: a propensity score matching based retrospective cohort study
Source: Front Endocrinol (Lausanne). 2024 Jan 25;15:1351991. doi: 10.3389/fendo.2024.1351991 (PMC10850314; doi:10.3389/fendo.2024.1351991)
Supplement: Supplementary file 1 [file Table_1.docx]

Supplementary Material

| **Group** |  | **NC** | **OI** | | **IVF** | | |
| --- | --- | --- | --- | --- | --- | --- | --- |
|  |  | N=47,348 | N=539 | *p* | N=3,285 | *p* | |
|  |  | n (%) | n (%) | Compare to NC | n (%) | Compare to NC | Compare  to OI |
| **Maternal characteristics** | | | | | | | |
| Age (y) | <30 | 19614 (41.4) | 229 (42.5) | <0.001 | 487 (14.8) | <0.001 | <0.001 |
|  | 30-34 | 18863 (39.8) | 264 (49.0) |  | 1507 (45.9) |  |  |
|  | ≥35 | 8871 (18.7) | 46 (8.5) |  | 1291 (39.3) |  |  |
| Pre-BMI (kg/m2) | <18.5 | 6345 (13.4) | 41 (7.6) | <0.001 | 265 (8.1) | <0.001 | <0.001 |
|  | ≥18.5＜24 | 34575 (73.0) | 349 (64.7) |  | 2256 (68.7) |  |  |
|  | ≥24＜28 | 3871 (8.2) | 74 (13.7) |  | 430 (13.1) |  |  |
|  | ≥28 | 2316 (4.9) | 74 (13.7) |  | 266 (8.1) |  |  |
|  | missing | 241 (0.5) | 1 (0.2) |  | 68 (2.1) |  |  |
| Gravidity | 1 | 22520 (47.6) | 317 (58.8) | <0.001 | 1695 (51.6) | <0.001 | <0.05 |
|  | 2 | 13618 (28.8) | 122 (22.6) |  | 871 (26.5) |  |  |
|  | ≥3 | 10721 (22.6) | 90 (16.7) |  | 672 (20.5) |  |  |
|  | missing | 489 (1.0) | 10 (1.9) |  | 47 (1.4) |  |  |
| Education level | ≤high school | 3519 (7.4) | 40 (7.4) | ns | 459 (14.0) | <0.001 | <0.001 |
|  | university education | 33936 (71.7) | 401 (74.4) |  | 2294 (69.8) |  |  |
|  | postgraduate  education | 9758 (20.6) | 97 (18.0) |  | 493 (15.0) |  |  |
|  | missing | 135 (0.3) | 1 (0.2) |  | 39 (1.2) |  |  |
| Han Chinese |  | 46514 (98.2) | 530 (98.3) | ns | 3233 (98.4) | ns | ns |
| Anemia |  | 16278 (34.4) | 168 (31.2) | ns | 995 (30.3) | <0.001 | ns |
| GDM |  | 6218 (13.1) | 118 (21.9) | <0.001 | 740 (22.5) | <0.001 | ns |
| Hypertension |  | 2610 (5.5) | 55 (10.2) | <0.001 | 384 (11.7) | <0.001 | ns |
| ICP |  | 351 (0.7) | 3 (0.6) | ns | 20 (0.6) | ns | ns |
| Preeclampsia  /eclampsia |  | 1147 (2.4) | 23 (4.3) | <0.05 | 175 (5.3) | <0.001 | ns |
| Placental problems |  | 1050 (2.2) | 11 (2.0) | ns | 170 (5.2) | <0.001 | <0.01 |
| **Neonatal characteristics** | | | | | | | |
| Male (%) |  | 24424 (51.6) | 262 (48.6) | ns | 1682 (51.2) | ns | ns |
| Jaundice (%) |  | 1031 (2.2) | 12 (2.2) | ns | 83 (2.5) | ns | ns |
| NICU (%) |  | 601 (1.3) | 7 (1.3) | ns | 56 (1.7) | <0.05 | ns |
| Preterm birth | <32 weeks | 179 (0.4) | 3 (0.6) | ns | 29 (0.9) | <0.001 | ns |
|  | <34 weeks | 411 (0.9) | 8 (1.5) | ns | 58 (1.8) | <0.001 | ns |
|  | <37 weeks | 2511 (5.3) | 43 (8.0) | <0.01 | 233 (7.1) | <0.001 | ns |
| Low birth weight | <1500 g | 129 (0.3) | 5 (0.9) | <0.05 | 21 (0.6) | <0.01 | ns |
|  | < 2500 g | 1363 (2.9) | 27 (5.0) | <0.01 | 125 (3.8) | <0.01 | ns |
| SGA |  | 1293 (2.7) | 18 (3.3) | ns | 86 (2.6) | ns | ns |

**sTable 1** Maternal and neonatal characteristics before PSM

Abbreviations: ns: no significance; GDM: Gestational diabetes mellitus; ICP: intrahepatic cholestasis of pregnancy; NICU: neonatal intensive care unit; SGA: small for gestational age
